# Duodenal ulcer penetration into the superior mesenteric artery after percutaneous transluminal angioplasty and stent placement for acute mesenteric ischemia: report of a case

**DOI:** 10.1007/s00595-013-0557-x

**Published:** 2013-05-17

**Authors:** Akira Ouchi, Masatoshi Isogai, Toru Harada, Yuji Kaneoka, Keitaro Kamei, Atsuyuki Maeda

**Affiliations:** Department of Surgery, Ogaki Municipal Hospital, 4-86 Minaminokawa-cho, Ogaki, Gifu 503-8502 Japan

**Keywords:** Acute mesenteric ischemia, Percutaneous transluminal angioplasty, Stent placement

## Abstract

A 78-year-old male presented with the chief complaints of abdominal pain and vomiting. Contrast-enhanced computed tomography and abdominal angiography showed occlusion of the superior mesenteric artery due to thrombosis, and emergency percutaneous transluminal angioplasty and stent placement were carried out. Two months later, stent thrombosis developed, and a second stent was placed. Eight months later, he complained of general fatigue and anorexia. Gastrointestinal endoscopy revealed a duodenal ulcer at the third portion close to the superior mesenteric artery. Thirteen days after conservative management, duodenal ulcer penetration into the superior mesenteric artery with subsequent air embolism developed, and the patient died of multiple organ failure.

## Introduction

Acute mesenteric ischemia (AMI) is a life-threatening disease. Despite the recent advances in the medical diagnoses and treatments, AMI still has a poor prognosis, with in-hospital mortality rates of 59–93 % because of the non-specific symptoms and signs of the disease [1]. Even in surviving patients, the long-term prognosis is poor due to short-bowel syndrome resulting from massive enterectomy.

Open surgical treatment with superior mesenteric artery (SMA) bypass or embolectomy has been the standard treatment for AMI, but the use of percutaneous transluminal angioplasty (PTA) and stent placement has increased in frequency. This is a less-invasive therapy than open surgical treatment, and it enables physicians to proceed immediately from diagnosis to treatment. According to Schermerhorn et al., PTA and stent placement “may be useful in selected patients with AMI and appropriate anatomy” [2].

PTA and stent placement is an effective treatment for AMI, but there are specific complications associated with the procedure. For example, it has been reported that patients who undergo PTA and stent placement for AMI have a potential risk of mesenteric artery complications, including distal mesenteric embolization, branch perforation, dissection, stent dislodgement, stent thrombosis and stent fracture [3, 4]. We herein emphasize the potential risk associated with stent placement in the SMA, particularly when it feeds many branches to the intestine by reporting a serious and previously unpublished complication of duodenal ulcer penetration into the SMA.

## Case report

**Fig. 1:**
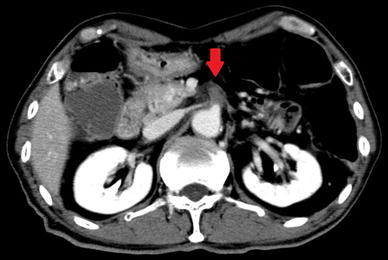
Contrast-enhanced computed tomography showed occlusion 3 cm across from the origin of the SMA (*down arrow*)

A 78-year-old Japanese male presented at our clinic with left lower quadrant pain and vomiting. He had undergone distal pancreatectomy and lymph node dissection for pancreatic body cancer 10 years earlier, and he also had histories of diabetes mellitus and hyperlipidemia. There was tenderness in the left lower quadrant of the abdomen without signs of peritonitis. Complete blood counts showed a high number of white blood cells (WBC; 19750/μL) and a blood biochemistry analysis showed elevated levels of lactase dehydrogenase (LDH; 283 IU/L), creatine phosphokinase (CPK; 558 IU/L) and C-reactive protein (CRP; 9.64 mg/L). The fibrin degradation products (5.6 μg/mL) and D-dimer (1.9 μg/mL) levels were also elevated. Contrast-enhanced computed tomography (CECT) showed occlusion 3 cm across from the origin of the SMA (Fig. 1). The distal blood flow was maintained by collateral blood circulation, and there was no finding suggestive of necrosis of the intestine. We made the diagnosis of AMI and carried out emergency abdominal angiography.

**Fig. 2:**
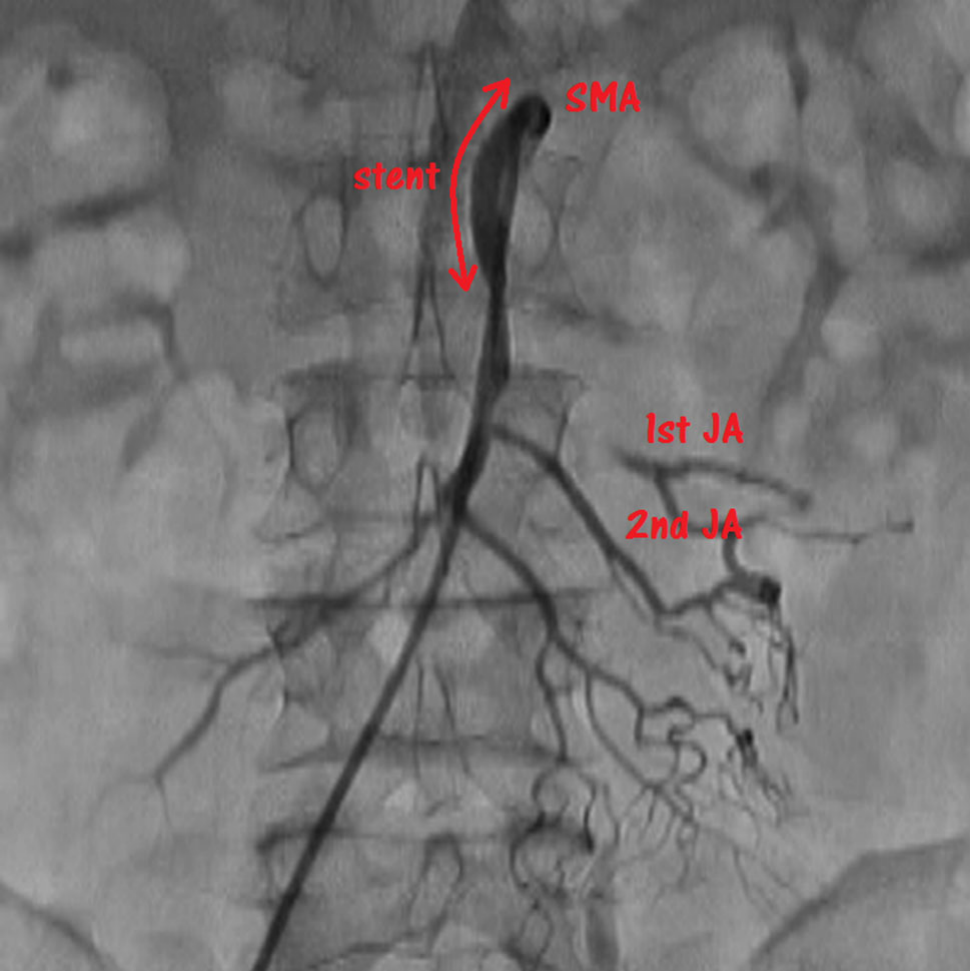
Angiography during the first operation showed that the first jejunal artery (*JA*) was occluded at its origin, and the marginal arteries of the first JA were maintained through collateral blood circulation from the second JA

Abdominal angiography by the right femoral approach showed that the SMA was occluded at its origin due to thrombosis resulting from atherosclerotic disease. We performed thrombectomy (Thrombuster II^®^, Kaneka Corp. Japan) and obtained recanalization. We also placed a self-expanding metallic stent (Zilver^®^ 7/40 mm, Cook Medical USA) into the SMA for residual stenosis. The first jejunal artery (JA) was occluded at its origin, and marginal arteries of the first JA were maintained through collateral blood circulation from the second JA (Fig. 2). The patient’s symptoms disappeared after the operation. We started to administer aspirin at 100 mg/day from the day after the operation to prevent stent thrombosis. He made satisfactory progress and was discharged from the hospital on post-admission day 23.

**Fig. 3:**
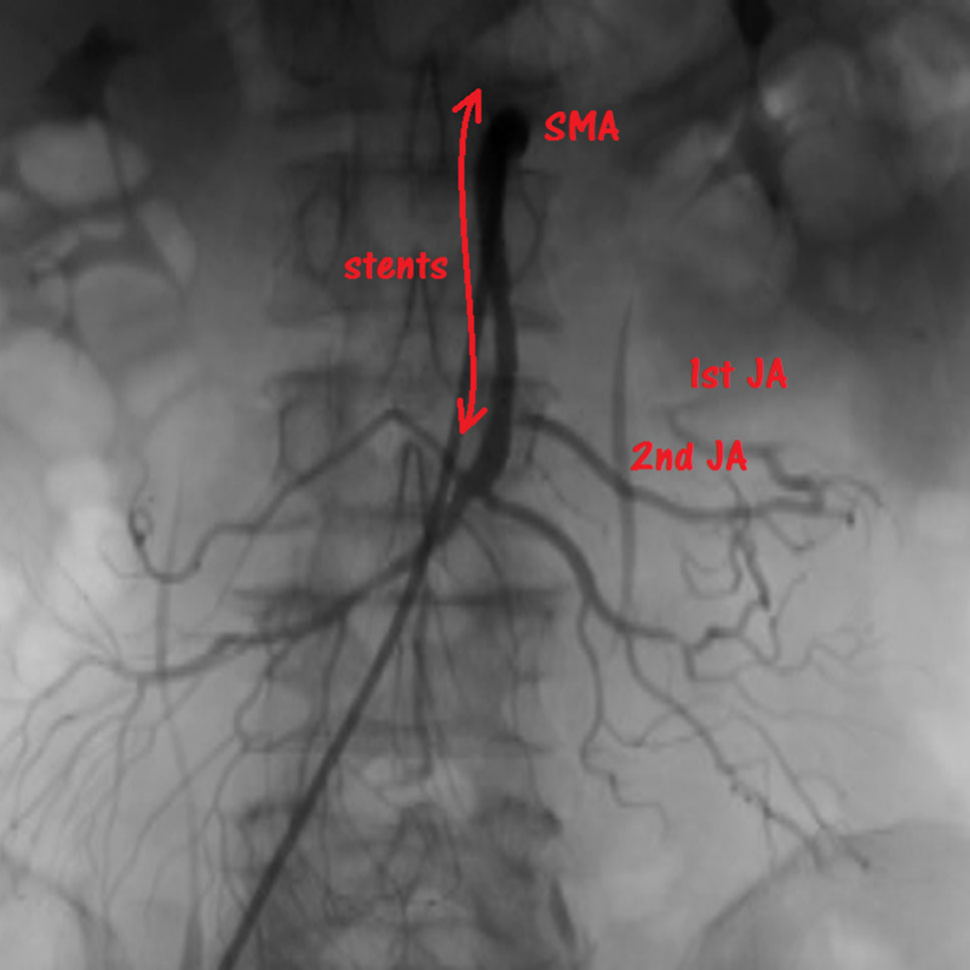
Angiography during the second operation showed that the blood flow of the second jejunal artery (*JA*) was good, but that it had decreased in the marginal arteries of the first JA

Two months later, he returned to our clinic with abdominal pain. CECT showed stent thrombosis of the SMA. We carried out emergency abdominal angiography again, which showed stent occlusion due to thrombosis. We performed thrombectomy and secondary stent placement (Zilver^®^ 4/60 mm) into the distal side of the previous stent across the origin of the second JA for residual stenosis, because we could not perform SMA bypass or embolectomy because of his past history of distal pancreatectomy and poor performance status. The blood flow of the second JA was good, but had decreased in the marginal arteries of the first JA (Fig. 3). We started to administer clopidogrel (at 75 mg/day) and warfarin, in addition to aspirin. He made satisfactory progress and was discharged from hospital on post-admission day 17.

**Fig. 4:**
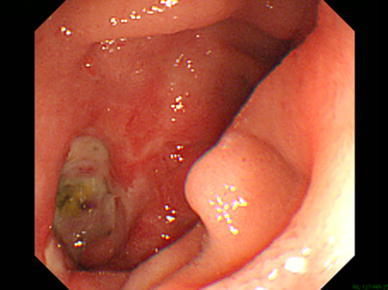
Gastrointestinal endoscopy showed a duodenal ulcer at the third portion 8 months later

**Fig. 5:**
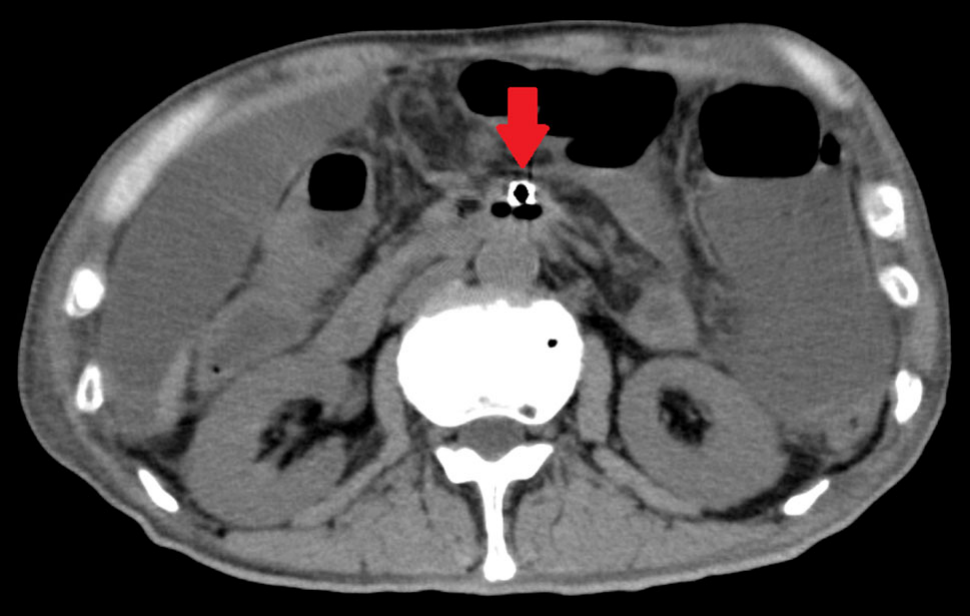
Computed tomography showed an air embolism of the SMA (*down arrow*), massive abdominal fluid collection and extensive mesenteric ischemia

Eight months later, he returned to our clinic again with general fatigue, anorexia and melena. His complete blood count showed a low hemoglobin level (7.2 g/dL). Gastrointestinal endoscopy showed a duodenal ulcer at the third portion (Fig. 4). We performed conservative management with fasting and a proton pump inhibitor. Thirteen days after admission, severe abdominal pain developed with signs of peritonitis, which spread to the entire abdomen. The complete blood count and blood biochemistry analysis showed a high WBC (12610/μL) and elevations of LDH (483 IU/L), CPK (467 IU/L) and CRP (24.16 mg/dL). Computed tomography showed an air embolism of the SMA, massive abdominal fluid collection and extensive mesenteric ischemia (Fig. 5). We diagnosed AMI due to air embolism of the SMA from a duodenal ulcer penetration into the SMA. At this time, his general condition was already too poor to tolerate open surgery, so we did not perform an operation. He died 18 days after admission.

## Discussion

PTA and stent placement for AMI has rapidly spread around the world during the last decade[5], based on recent studies reporting favorable short-term outcomes with revascularization rates of 73–100 % and in-hospital mortality rates of 24–36 % [6–10]. With the availability of embolic protection devices and low-profile stent systems, mesenteric stent placement has emerged as a useful treatment strategy [11]. However, nearly 40 % of patients with mesenteric stent placement were reported to develop in-stent restenosis, half of whom required reintervention [12].

Furthermore, mesenteric stent placement remains controversial in terms of its complications. Here, we reported the development of duodenal ulcer penetration into the SMA with subsequent air embolism, which developed after PTA and stent placement for AMI. To the best of our knowledge, this is the first report of a serious complication of PTA and stent placement.

One possible cause of the duodenal ulcer related to mesenteric stent placement is a blood flow disturbance in the duodenum wall. Angiography during the first operation showed that the first JA was occluded at its origin, but that the marginal arteries of the first JA were maintained through collateral blood circulation from the 2nd JA. At the second operation, however, the additional stent placed across the origin of the second JA may have disturbed or occluded the collateral blood circulation from the second JA to the first JA, leading to reduced blood flow to the third portion of the duodenum. Moreover, there might have been prior disruption of the collateral vessels due to the distal pancreatectomy for a pancreatic tumor. In our case, the duodenal ulcer occurred some time after stent placement. At the second operation, the blood flow of the second JA had decreased, but was maintained at a sufficient level. The third portion of the duodenum would also be nourished through the gastroduodenal artery (GDA)-inferior pancreatoduodenal artery (IPDA) communication for a while. Although it is mere conjecture due to the lack of angiographic evidence, the duodenal ulcer might have been associated with the formation of a new microthrombus distal to the first JA.

Another possibility is mechanical irritation by the stents. One of the most serious complications after Y-graft placement for an abdominal aortic aneurysm is an aortoenteric fistula. Thus, chronic duodenal wall irritation by mesenteric stent placement might also be another possible cause of duodenal ulcer formation. However, gastrointestinal endoscopy did not show any ischemic changes of the duodenum. We also consider that the duodenal ulcer might have occurred due to multiple causes, including blood flow disturbance and mechanical irritation.

Brown et al. [11] reported that mesenteric stenting is associated with early restenosis and recurrent symptoms requiring secondary procedures. But later, Block et al. reported that the results after endovascular and open surgical revascularization of AMI were favorable, in particular, among the endovascularly treated patients [12]. The superiority of mesenteric stenting remains controversial. In our case, the patient’s performance status was poor, so we selected the less-invasive therapy.

Our case suggests a possible role of mesenteric stent placement in duodenal ulcer formation. Patients with mesenteric stent placement should be followed closely, especially those with reduced duodenal blood flow or with repeated interventions due to an ongoing high risk for open surgery. Long-term follow-up with a larger number of patients is necessary to ultimately determine the role of mesenteric stent placement in duodenal ulcer formation.
